# Kin5 Knockdown in *Tetrahymena thermophila* Using RNA*i* Blocks Cargo Transport of Gef1

**DOI:** 10.1371/journal.pone.0004873

**Published:** 2009-03-17

**Authors:** Aashir Awan, Aaron J. Bell, Peter Satir

**Affiliations:** Department of Anatomy & Structural Biology, Albert Einstein College of Medicine, Bronx, New York, United States of America; Emory University, United States of America

## Abstract

A critical process that builds and maintains the eukaryotic cilium is intraflagellar transport (IFT). This process utilizes members of the kinesin-2 superfamily to transport cargo into the cilium (anterograde transport) and a dynein motor for the retrograde traffic. Using a novel RNA*i* knockdown method, we have analyzed the function of the homodimeric IFT kinesin-2, Kin5, in *Tetrahymena* ciliary transport. In RNA*i* transformants, Kin5 was severely downregulated and disappeared from the cilia, but cilia did not resorb, although tip structure was affected. After deciliation of the knockdown cell, cilia regrew and cells swam, which suggested that Kin5 is not responsible for the trafficking of axonemal precursors to build the cilium, but could be transporting molecules that act in ciliary signal transduction, such as guanine nucleotide exchange proteins (GEFs). Gef1 is a *Tetrahymena* ciliary protein, and current coimmunoprecipitation and immunofluorescence studies showed that it is absent in regrowing cilia of the knockdown cells lacking ciliary Kin5. We suggest that one important cargo of Kin5 is Gef1 and knockdown of Kin5 results in cell lethality.

## Introduction

Recent studies suggest that *Tetrahymena* cilia may contain evolutionary precursors of important metazoan signaling pathways [1], [2], [3]. From studies on *Chlamydomonas* [4], it has become apparent that most motile protistan and non-motile sensory metazoan cilia, including human primary cilia, are built by intraflagellar transport (IFT) whose molecular constituents are largely conserved. However, the details of ciliary cargo transport, especially of transport of signaling and membrane components into the cilium, although critical for sensory cilia function, have not yet been fully elucidated. It has been shown that certain axonemal components are transported in the cilium by a heterotrimeric kinesin-2 [5], which in *Tetrahymena* is probably represented by the motor proteins, Kin1 and Kin2 [6] (protein notation taken from kinesin literature standards). In previous findings, we characterized Kin5 as a new, probably essential, IFT motor for intraciliary transport in *Tetrahymena* [7], which could be involved in the transport of certain membrane components as part of signaling systems. We cloned the 2508 bp coding region of Kin5 and identified it as a member of the kinesin-2 subfamily, whose closest phylogenetic neighbors were Osm3, a *C. elegans* homodimeric IFT motor protein that is responsible for building the distal segments of sensory cilia in that organism [8], [9], [10] and Kif17, originally identified as a mammalian neuronal protein involved in the transport of NMDA receptor [11] and later shown also to transport ciliary receptors [12]. Northern and western blots localized Kin5 to the cilium. With immunofluorescence, Kin5 was shown to be present in a punctate pattern along the cilium, colocalizing with orthologs of *Chlamydomonas* IFT complex proteins [13], including members of both IFT complex A (IFT 139/140), the complex probably involved in retrograde transport and complex B (IFT 57, 81, 88, 172) the anterograde transport complex necessary for building the axoneme [14]. Because permeabilization for immunolocalization was performed in the presence of AMP-PNP, it is likely that Kin5 is attached to both the doublet microtubules and the transport complexes.

We wanted to explore the function of Kin5 as a *Tetrahymena* IFT motor possibly involved in the intraciliary transport of membrane-associated signaling molecules toward the cilium tip. To do this, we proposed to alter the amount of Kin5 produced in the cell and observe how the phenotype is altered. Typically, *Tetrahymena* contains a somatic macronucleus and a germline micronucleus. Using biolistic transformation and a mutant construct based on the work of Cassidy-Hanley et al. [15], we were able to disrupt the macronuclear *KIN5* locus. However, after phenotypic assortment, we failed to generate a somatic knockout, which indicated that this was probably an essential gene whose knockout would lead to lethality. We then decided to investigate whether RNA*i* production for gene knockdown of *KIN5* was feasible in *Tetrahymena*.

In this study, we define a successful technology for *KIN5* knockdown by an inducible short hairpin RNA (*sh*RNA), with relevant conditions and controls. We characterize the *KIN5* knockdown phenotype by cell survival studies to confirm that *KIN5* is an essential gene, and we discuss the difficulties this presents in the knockdown experiments. We present data to indicate that Kin5 has novel intraciliary transport cargos, some probably related to the placement of membrane and signaling molecules within the cilium, rather than to the transport of components of the motile axoneme, and we discuss similarities and possible differences between the functions of Kin5 and Osm3 or Kif17.

## Results

### Construction of pK5KOAs.40/pInv2 and cell transformation

**Figure 1:**
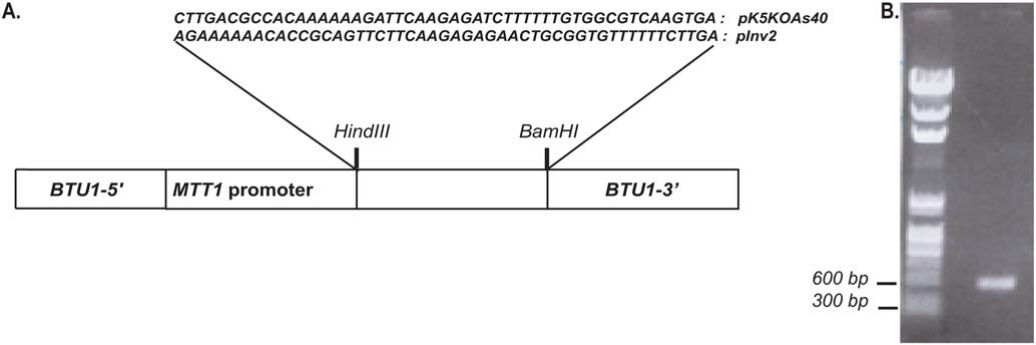
pK5KOAs40/pInv2 Vector Constructs. A. Schematic diagram showing nucleotide sequences of *K5KOAs.40* and *Inv2* for *sh*RNA inserted into the MTT1-containing vector. B. PCR verification of *KO* insert. Left lane: marker DNA; Right lane: Expected PCR product on genomic DNA based on MTTI.s and BTU1.a primers for the *KO* insert. An identical band was seen with the *Inv2* insert for Inv2 cells.

Our initial goal was to deplete Kin5 from the cells by integrating an inducible *sh*RNA into the genome. *T. thermophila* expresses two redundant β-tubulin genes, *BTU1* and *BTU2*. The pMTT1-BICH3 construct designed by Gorovsky's laboratory is able to target the *IAG48[G1]* sequence behind the powerful inducible metallothionein *MTT1* promoter to the *BTU1* coding region by biolistic transformation [16]. The *IAG48[G1]* sequence can be replaced by other sequences of interest that will be transcribed when a heavy-metal divalent cation such as Cd^2+^ is added. To introduce an inducible RNA*i* construct into the macronuclear genome, we replaced the *IAG48[G1]* sequence with a sequence which when transcribed would fold to produce a short double-stranded RNA. A short sequence (*K5KOAs.40*) based on 19 nucleotides from the *KIN5* stalk coding region was chosen, which was unique to the *KIN5* gene as verified by a blast search of the *Tetrahymena* genome (http://www.tigr.org/tigr-scripts/tgi/T_index.cgi?species=t_thermophila). We then designed a construct containing the coding sequence, a loop of nine bases, the antisense sequence of the coding region and a termination codon (Figure 1A). We hypothesized that this construct, *K5KOAs.40*, would transcribe into a short double-stranded RNA that would act in an RNA*i* mechanism to suppress the expression of *KIN5*. A similar scheme was used to construct *Inv2* which employed the same 19-nt sequence, but in the inverse orientation (Figure 1A), a type of scrambled sequence that was routinely used as a control for RNA*i* experiments. Using the HindIII and BamHI sites, the constructs were ligated into the modified MTT1 plasmid, here pK5KOAs.40 and pInv2, respectively and used for biolistic transformation as described by Cassidy-Hanley et al. [15].

CU522 cells contain a point mutation in the *BTU1* gene, which renders them taxol-sensitive [16]; replacement of the *BTU1* gene upon incorporation of the plasmid transforms them to taxol-resistant. After transformation of the CU522 cells with the pK5KOAs.40 or pInv2 constructs, the cells were kept at 20 µM paclitaxel, for phenotypic assortment, selecting for more and more copies of the *K5KOAs.40* construct in the macronucleus as growth proceeded for a few months [17]. Using BTU1.a and MTT1.s primers in a PCR reaction, we verified that the *K5KOAs.40* and *Inv2* constructs were integrated into the correct loci (Fig 1B). After allowing for phenotypic assortment, single-cell isolates were grown up to stationary phase for further experimentation.

### Induction of *sh*RNA via the *MTT1* promoter

The metallothionein gene, *MTT1*, responds to stress and to heavy metals. In particular, Cd^2+^ serves to induce transcription by the promoter greater than 200-fold over control conditions [18]. In order to test whether our construct would work to knockdown *KIN5* by an RNA*i* mechanism and to define the time course of knockdown in *Tetrahymena*, we performed RT-PCR in a linear response range for qualitative analysis on samples following the addition of CdCl_2_, initially at 5.0 µg/ml concentration. We saw a much stronger response to Cd^2+^ when the clones were grown in 10 mM Tris-HCl, pH 7.5, as compared to 2× proteose peptone (2XPP). All further experiments were performed in Tris under starvation conditions. A non-ciliary control RNA (*PGM1*) was examined simultaneously with *KIN5* RNA.

**Figure 2:**
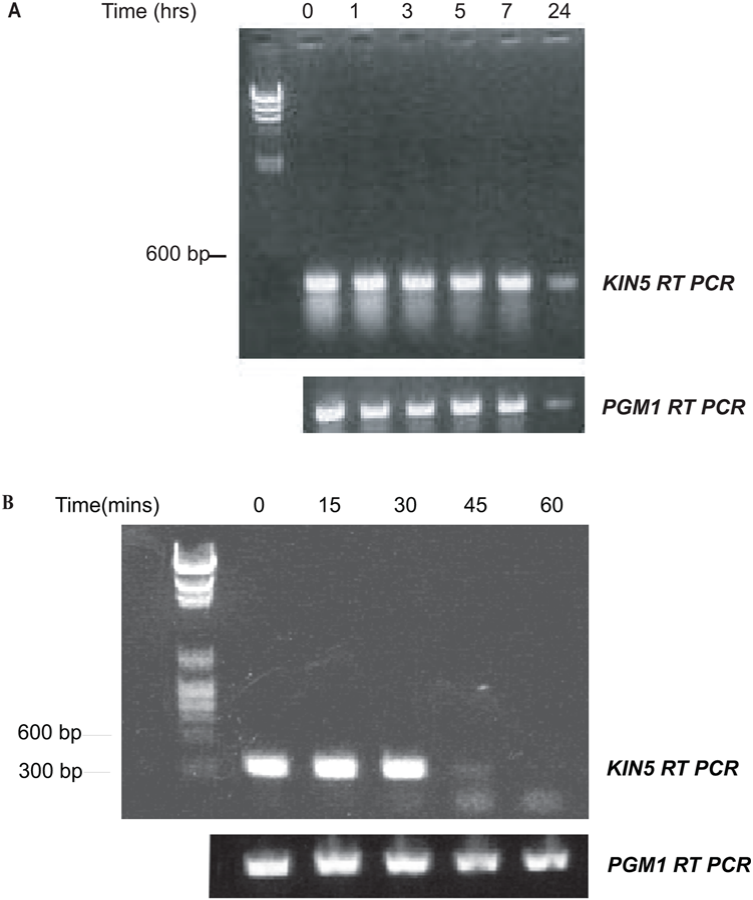
Stability of *KIN5* and *PGM1* messages. A. CU522 cells grown in starvation conditions+5.0 µg/ml Cd^2+^ prior to transformation showing comparable relative stabilities of the *KIN5* and *PGM1* mRNA. B. Time course of degradation of *KIN5* message after *sh*RNA induction in K5KOAs.40 cells using 5.0 µg/ml Cd^2+^. RT-PCR products resolved on a 1% agarose gel. Left lane: DNA markers. The *KIN5* message decreases at 45 min post-induction and is eliminated at 60 min. The *PGM1* message remains constant.

When cells were exposed to 5.0 µg/ml Cd^2+^ in the absence of transformation, both *KIN5* RNA and *PGM1* RNA remained qualitatively unchanged for 7 h and both messages persisted for at least 24 h (Figure 2A).

**Figure 3:**
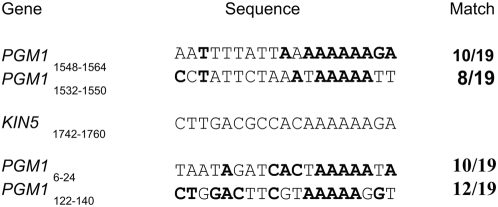
Sequence Comparison of *K5KOAs.40* vs. *PGM1*. The indicated *PGM1* coding regions show high degree of homology to the 19 nt (positions 1742–1760) chosen for *KIN5 sh*RNA yet *KIN5 sh*RNA does not affect *PGM1*.

Figure 2B shows that after Cd^2+^ addition, the *KIN5* message remained unchanged only in the samples taken 0, 15 and 30 min post-induction. By 45 min, the message greatly decreased and finally by 60 min, the message was no longer seen. There was no change in the *PGM1* message in the KO cells for the entire time period, even though there is identity between the 19-nt *KIN5* region and various stretches of the *PGM1* gene in as many as 12 of the 19-nt positions (Figure 3). We conclude that the transcribed *K5KOAs.40* construct specifically eliminated *KIN5* RNA within 1 hour of induction, which suggests that the construct strategy was successful, probably working as hypothesized, via *sh*RNA transcription and an RNA*i* mechanism.

### Optimization of [Cd^2+^]

**Figure 4:**
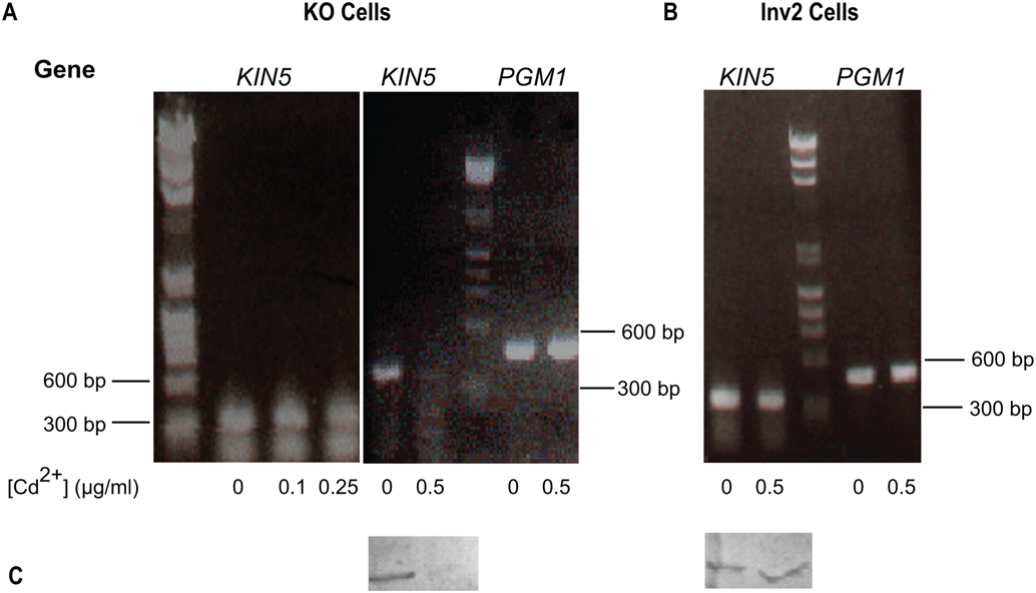
Optimization of *KIN5 sh*RNA. A. Degradation of *KIN5* message after *sh*RNA induction in KO cells using 0–0.5 µg/ml Cd^2+^. Above: RT-PCR products resolved on a 1% agarose gel. At Cd^2+^ concentrations lower than 0.5 µg/ml, *KIN5* mRNA is stable for 24 h. After 24 h in 0.5 µg/ml Cd^2+^, *KIN5* mRNA is dramatically decreased, while *PGM1* is unaffected. B. Effect of 0.5 µg/ml Cd^2+^ on *KIN5* and *PGM1* messages in Inv2 cells. *KIN5* and *PGM1* mRNA levels remain unaffected after 24 h. DNA markers shown: lines indicate 600 and 300 bp. C. Effect of 0.5 µg/ml Cd^2+^ on Kin5 protein levels in KO and Inv2 cells. Corresponding KO (left) and Inv2 (right) cell homogenates 12 h post-induction at either 0 or 0.5 µg/ml Cd^2+^ and blotted with K5T1 Ab to Kin5. While the Kin5 protein is severely knocked down in the KO cells upon *sh*RNA induction, Kin5 levels remain unaffected in Inv2 cells under similar conditions.

Viability and motility could also be affected by Cd^2+^, in concentrations that could be toxic under starvation conditions. In 5.0 µg/ml Cd^2+^, the KO cell population was not viable much beyond 1 h. In untransformed and Inv2 transformed cells in this Cd^2+^ concentration, viability also dropped sharply within a few hours, so that less than 10% of the population persisted for 24 h. Many cells ruptured, presumably because of failure of osmoregulation, which is a non-specific effect of heavy metal poisoning [19]. We set about to find the optimal [CdCl_2_] that would elicit the RNA*i* response without affecting the control cell viability or causing the cells to rupture. We chose concentrations of 0, 0.1, 0.25, and 0.5 µg/ml CdCl_2_ and performed RT-PCR at 0, 6, and 24 h on KO and Inv2 cell populations. At 0 and 6 h, the *KIN5* message was present in similar amounts in all the samples. By 24 h, in KO cells, the *KIN5* message was unchanged in 0, 0.1 or 0.25 µg/ml Cd^2+^, but essentially absent in 0.5 µg/ml CdCl_2_, while the *PGM1* levels remained unaffected (Fig 4A). Both the *KIN5* and *PGM1* messages remained unaffected in Inv2 control cells from 0 to 24 h at 0.5 µg/ml Cd^2+^ (Figure 4B). The is confirmed by the loss of Kin5 protein in the KO cells upon cadmium induction whereas Kin5 levels remain unchanged in the Inv2 cells (Figure 4C)

**Figure 5:**
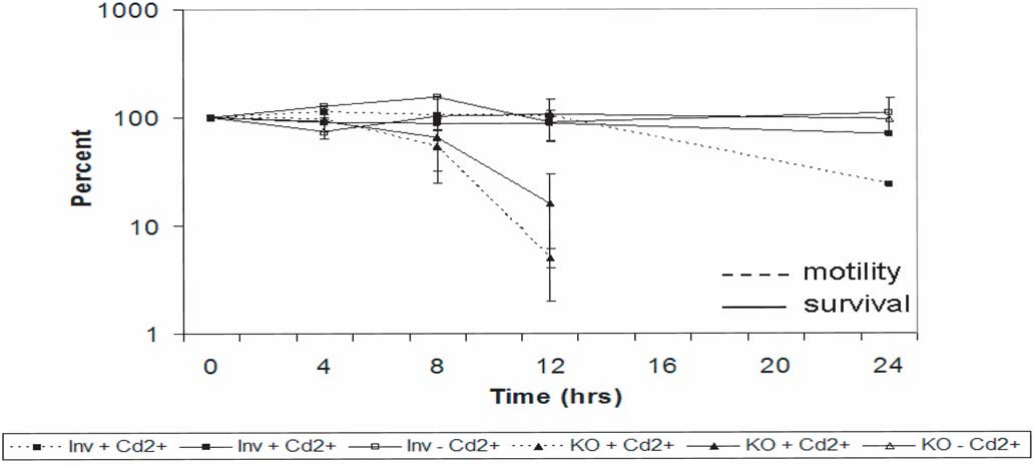
Effect of 0.5 µg/ml Cd^2+^ on K5KOAs.40 and Inv2 cell survival. Initial population indicated as 100%. Solid lines: survival measured by cell count. Dashed lines: Survival measured by cell motility. Essentially, all surviving cells in the culture are motile.

To show that Cd^2+^ was not toxic at 0.5 µg/ml, we measured cell survival with or without *KIN5* knockdown, compared to untreated controls. In cells containing the Inv2 construct with or without Cd^2+^, survival at 24 h decreased only slightly with addition of cadmium (Figure 5). This suggests that Cd^2+^ toxicity at this concentration was minimal. Cells containing the KO construct without Cd^2+^ also survived as expected, but when Cd^2+^ was added so that Kin5 was knocked down, only about 10% of the original population survived. For the initial 12 h, the time course of loss of viability as measured by cell count paralleled the loss of *KIN5* RNA and of survival measured by motility (Figure 5), implying that the reduced population under study remained viable. By 12 h, most of the KO cells were moving very slowly, while overall cell shape remained unchanged, nuclear morphology was normal and cilia were still beating. Taken together, these observations suggested that the *KIN5* knockdown phenotype produced loss of normal coordinated movement and then lethality, which is consistent with our earlier finding that *KIN5* is an essential gene. We concluded that most of the lethality seen at 12 h in the K5KOAs.40 population was not due to the presence of Cd^2+^, but rather to the loss of *KIN5* mRNA and its consequences and that, at this time, there are still enough viable cells for study. In further experiments, we used 0.5 µg/ml Cd^2+^ and 12 h exposure to study the effects of *KIN5* knockdown on Kin5 localization.

### Localization of Kin5

**Figure 6:**
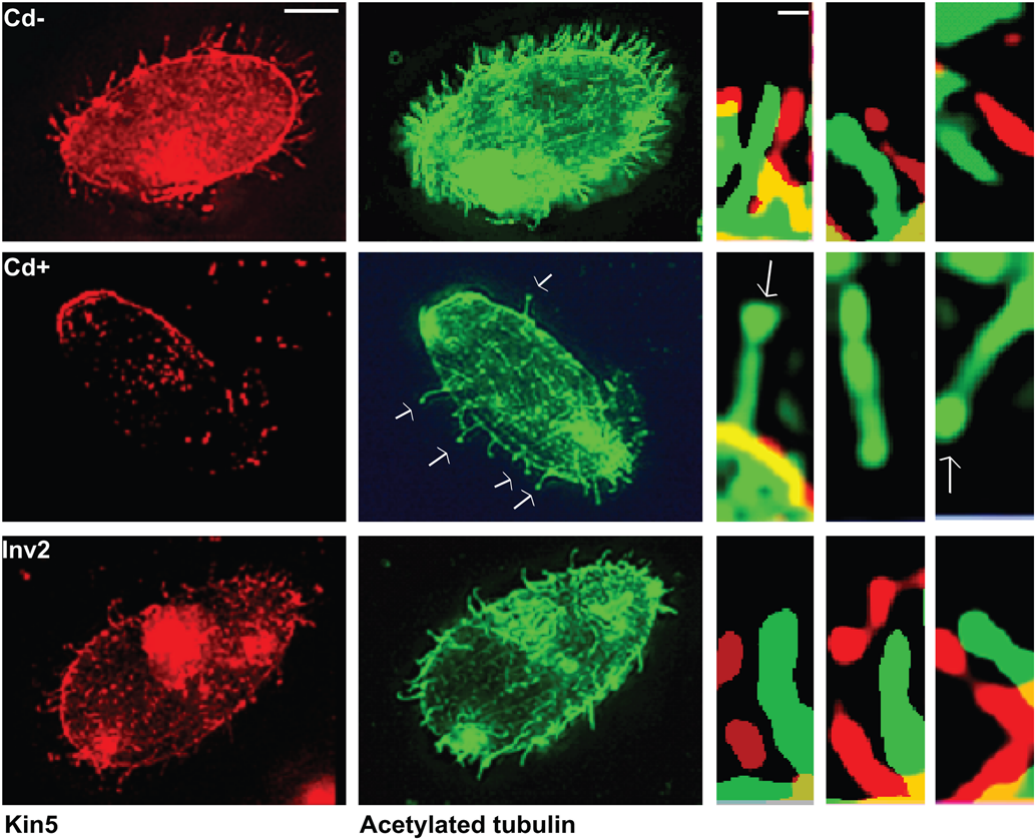
Kin5/tubulin immunofluorescence in cells without deciliation. Top panel: K5KOAs.40 cells with 0 µg/ml Cd^2+^; middle panel: K5KOAs.40 cells with 0.5 µg/ml Cd^2+^ (12 h); bottom panel: Inv2 cells with 0.5 µg/ml Cd^2+^ (12 h). Right panels: Enlarged cilia showing the presence or absence of punctate pattern of Kin5 antibody (red) localization offset from the continuous tubulin localization (green). Scale bars: (left) 10 µm; (right) 1 µm. In K5KOAs.40 cells treated with Cd^2+^, Kin5 fluorescence is missing in the cell body and the cilia.

From previous studies [7], Kin5 localized to cilia in a punctate pattern, which is consistent with activity as an intraciliary transport motor. To confirm that loss of *KIN5* RNA resulted in loss of Kin5 in cilia, we used a specific Kin5 Ab [7]. Immunofluorescence microscopy confirmed that after 12 h exposure to 0.5 µg/ml Cd^2+^, Kin5 was greatly reduced in K5KOAs.40 cells, while Kin5 in Inv2 cells was unaffected (Figure 4). The Kin5 signal completely disappeared from cilia upon exposure to CdCl_2_, although the cilia seemed otherwise unaffected. Some cilia, however, were found to have bulbous tips, as if tip elongation were blocked. In contrast, Kin5 remained in the cilia of Inv2 cells after 12 h in CdCl_2_ (Figure 6).

**Figure 7:**
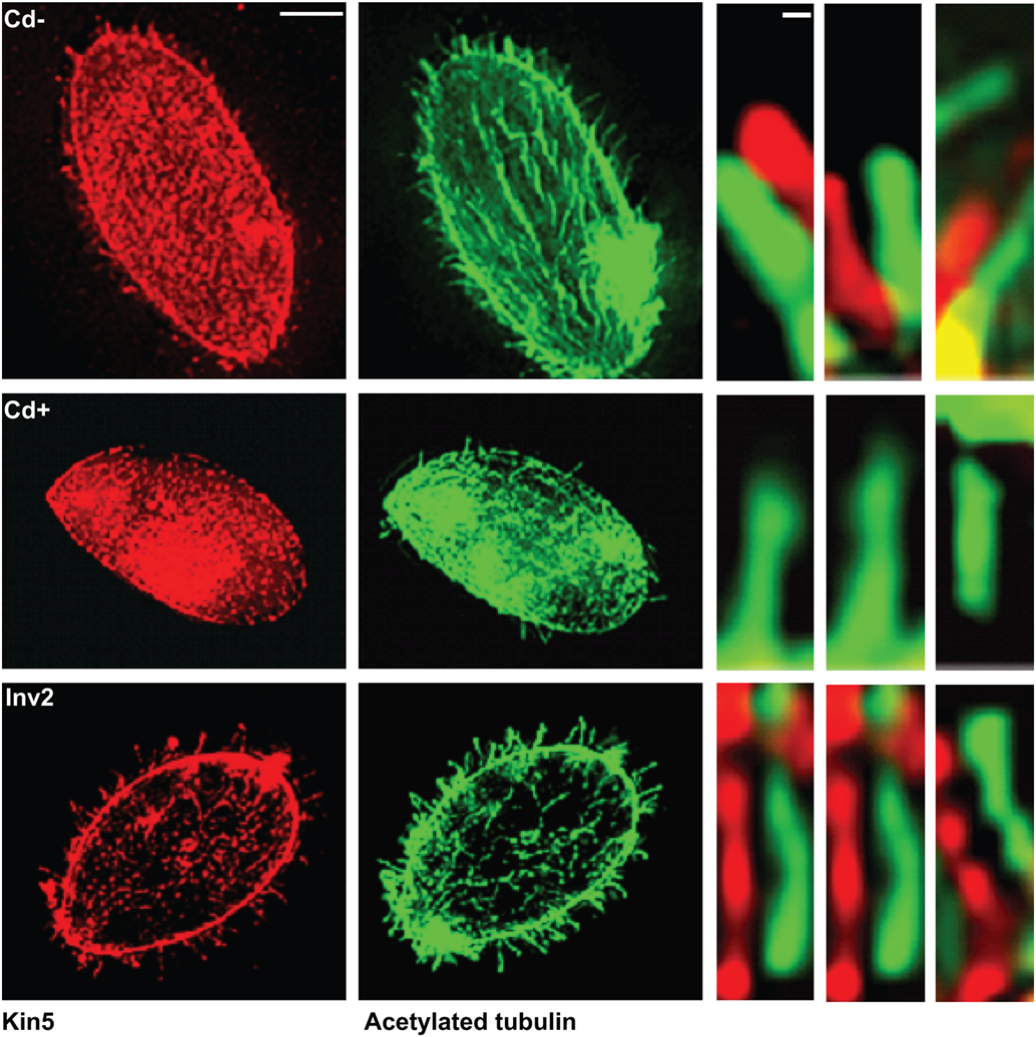
Kin5/tubulin immunofluorescence in cells after deciliation and ciliary regrowth for 2 h. Top panel: K5KOAs.40 cells with 0 µg/ml Cd^2+^; middle panel: K5KOAs.40 cells with 0.5 µg/ml Cd^2+^ (12 h); bottom panel: Inv2 cells with 0.5 µg/ml Cd^2+^ (12 h). Right panels: Enlarged cilia showing the presence or absence of punctate pattern of Kin5 antibody (red) localization offset from the continuous tubulin localization (green). Scale bars: (left) 10 µm; (right) 1 µm. After deciliation, Cd^2+^ treated-K5KOAs.40 cells regrow cilia without Kin5.

To address whether or not *KIN5* knockdown due to RNA*i* affects axonemal assembly and function, we deciliated the cells just prior to CdCl_2_ treatment. After deciliation, the cells were initially non-motile. In the absence of CdCl_2_, the K5KOAs.40 cells were able to regenerate cilia and begin swimming after 2 h. The newly formed cilia contained Kin5. After CdCl_2_ treatment, the deciliated cells still grew cilia, but fewer and perhaps of shorter length. The cells became motile, but although some cytoplasmic Kin5 staining could be seen, ciliary Kin5 was completely absent. In contrast, the Inv2 cells continued to show Kin5 localization in the cilia after deciliation and CdCl_2_ treatment (Figure 7).

### Gef1 Transport by Kin5

**Figure 8:**
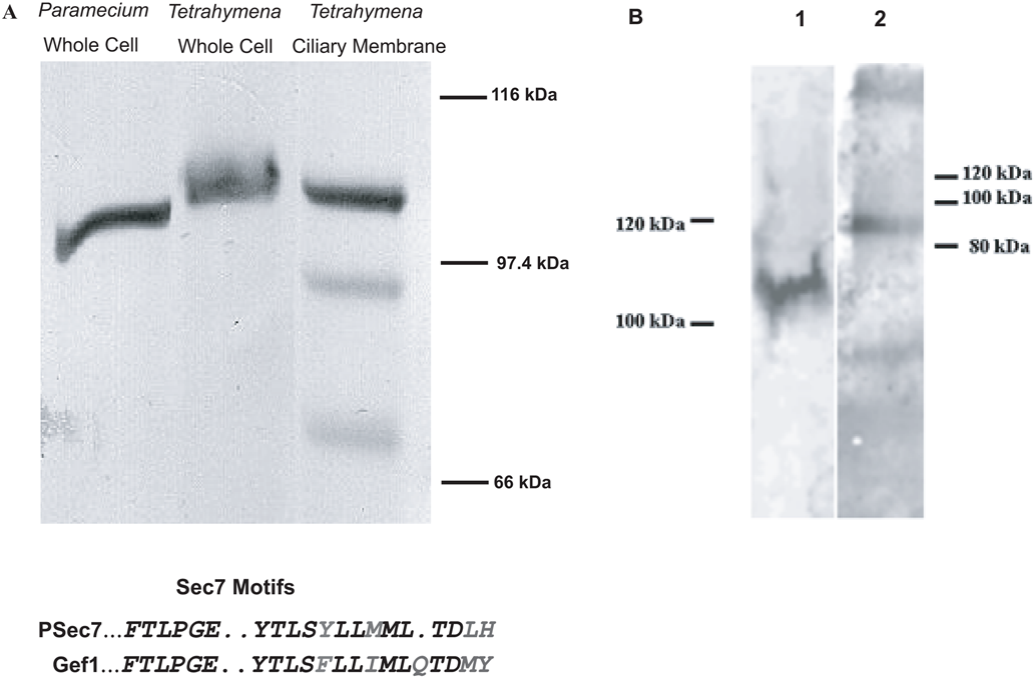
Gef1 is a cargo of Kin5. A. Immunoblot of Gef1. The Gef1 Ab identifies a ca. 100 kDa band (PSec7) from *Paramecium*, and an ortholog (Gef1) in *Tetrahymena* cell and ciliary membrane fractions. The Sec7 motif, which delineates a function in guanine nucleotide exchange, is found in both proteins (Grey letters indicate similarity). B. Co-immunoprecipitation of Kin5 and Gef1. 1: Kin5 immunoprecipitate probed with Gef1 Ab. 2: Gef1 immunoprecipitate probed with Kin5 Ab.

**Figure 9:**
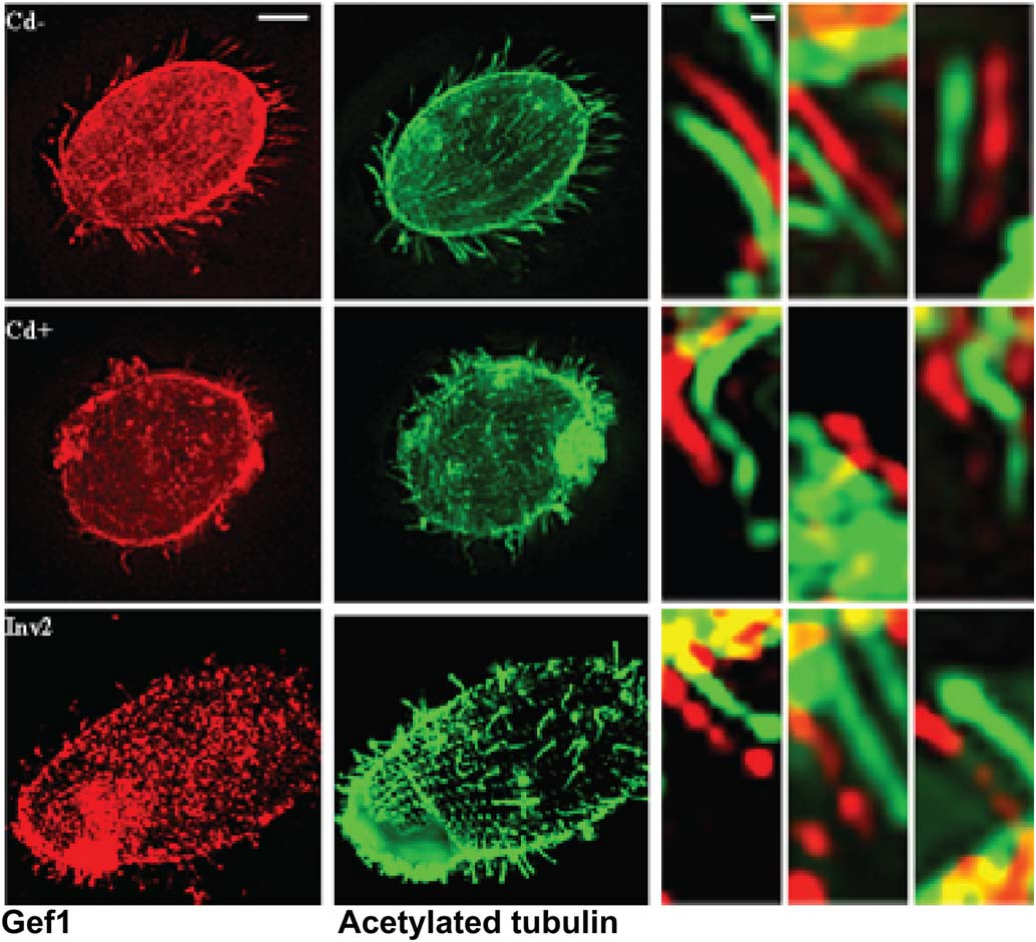
Gef1/tubulin immunofluorescence in cells without deciliation. Top panel: K5KOAs.40 cells with 0 µg/ml Cd^2+^; middle panel: K5KOAs.40 cells with 0.5 µg/ml Cd^2+^ (12 h); bottom panel: Inv2 cells with 0.5 µg/ml Cd^2+^ (12 h). Right panels: Enlarged cilia showing the presence or absence of punctate pattern of Gef1 antibody (red) localization offset from the continuous tubulin localization (green). Scale bars: (left) 10 µm; (right) 1 µm. In KO cells treated with Cd^2+^, unlike Kin5, Gef1 remains in the cilia.

**Figure 10:**
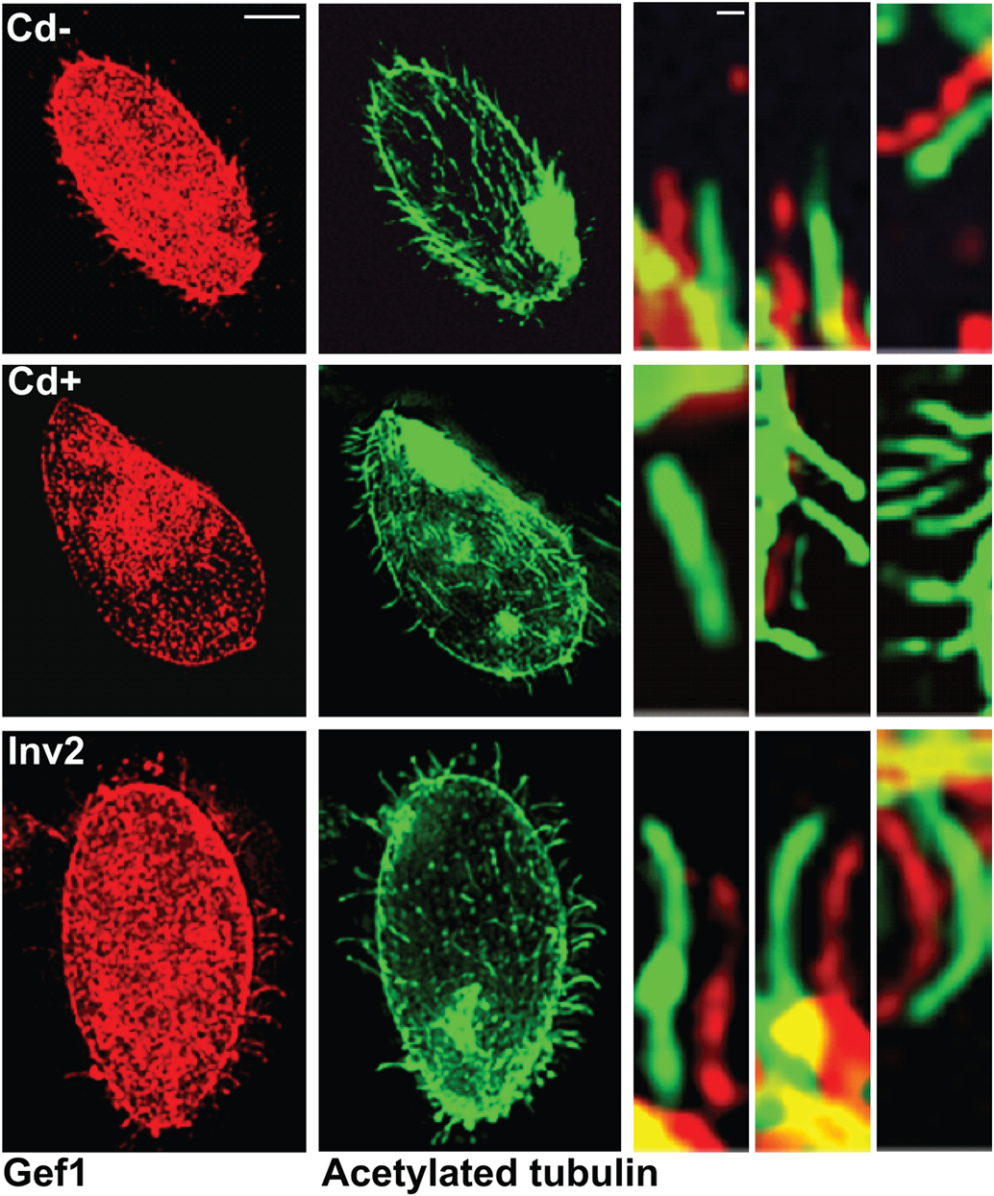
Gef1/tubulin immunofluorescence in cells after deciliation and ciliary regrowth for 2 h. Top panel: K5KOAs.40 cells with 0 µg/ml Cd^2+^; middle panel: K5KOAs.40 cells with 0.5 µg/ml Cd^2+^ (12 h); bottom panel: Inv2 cells with 0.5 µg/ml Cd^2+^ (12 h). Right panels: Enlarged cilia showing the presence or absence of punctate pattern of Gef1 antibody (red) localization offset from the continuous tubulin localization (green). Scale bars: (left) 10 µm; (right) 1 µm. In control cells, Gef1, like Kin5, returns to the cilia. In KO cells treated with Cd^2+^, Gef1, like Kin5, does not return to the cilium.

Kin5 has a close phylogenetic relationship to Kif17, a kinesin involved in the transport of NMDA-containing vesicles [11] and of cyclic nucleotide gated channels in mammalian ciliated cells [12] which suggested that Kin5 might also be involved in the transport of membrane proteins or signaling molecules. Gef1 is a *Tetrahymena* ciliary protein whose orthologue appears to be a guanine nucleotide exchange factor (GEF) of the Sec7 family, cloned in *Paramecium*. An antibody to the PH domain of the *Paramecium* protein (PSec7, [20]) recognized the corresponding protein fragment at ca. 100 kDa in *Tetrahymena* cilia, and therefore identified Gef1 (Figure 8). A pulldown using the Kin5 (K5T1) antibody also pulled down the Gef1 fragment, while in reverse, a pulldown of Gef1 using the PSec7 antibody also pulled down Kin5 (Figure 8), which suggests that Gef1 could be a cargo of Kin5. As a control, when the antibody was omitted, no bands were seen. If Gef1 was one such cargo of Kin5, transport of Gef1 should be disrupted in the K5KOAs.40 knockdown cells. We therefore examined Gef1 localization in KO and Inv2 cells before and after exposure to CdCl_2_. As anticipated, Gef1 identified by the PSec7 antibody localized along the cilium with Kin5 in the KO and Inv2 cells after 12 h if no Cd^2+^ was present and also in Inv2 cells when exposed to Cd^2+^ (Figure 9). Surprisingly, Gef1 was still present in the cilia of K5KOAs.40 cells after exposure to Cd^2+^, when Kin5 was no longer seen (Figure 9). This might occur if Kin5 transported Gef1 into the cilium and then released it along the ciliary membrane where Gef1 persisted even after ciliary Kin5 depletion. To test this hypothesis, we deciliated the K5KOAs.40 cells and examined the localization of Gef1 as cilia regrew in the absence vs. presence of Cd^2+^. In the absence of Cd^2+^, Gef1 and Kin5 both reappeared in the growing cilium, but in the presence of Cd^2+^, although the cilia regrew, neither Gef1 (Figure 10) nor Kin5 was present (Figure 7). We conclude that Gef1 is a likely Kin5 cargo that can be released from the transport apparatus to remain along the cilium.

## Discussion

### RNAi in *Tetrahymena*

For this study, we developed a method of knockdown of a specific macronuclear transcript in *Tetrahymena* by constructing an inducible *sh*RNA. We relied on previous methodology [21]. We chose a 19 nt sequence specific to the gene of interest, *KIN5*, and linked the sense and antisense strands by a short hairpin which would be excised during processing [22]. We placed this behind the metallothionein promoter and used biolistic transformation to insert the construct into the genome. After selection for phenotypic assortment and addition of an inducer (Cd^2+^), we could demonstrate specific knockdown of the mRNA of interest, while a control transcript was unaffected. The inverse construct from the 19-nt sequence had no effect. This methodology should be applicable to *sh*RNA production for knockdown of other genes of interest in *Tetrahymena*. A similar conclusion was reached by Howard-Till and Yao [23].

In our experiments, we transformed and selected cells grown to stationary phase in growth medium (proteose peptone), but for *sh*RNA induction, following Nilsson [19], we moved the cells into a simplified buffer that does not support division and yielded cells that were more sensitive to Cd^2+^. Presumably, under growth conditions, more MTT1 protein will be synthesized to transport Cd^2+^ out of the cytoplasm. We found that exposure to 5.0 µg/ml Cd^2+^ produced a knockdown, but was highly toxic, leading to cell death within a few hours, even in control cells. Cells transformed with an RNA*i* control construct (*Inv2*) at an order of magnitude lower concentration of Cd^2+^ (0.5 µg/ml) were unaffected for at least 12 h, suggesting that there was a little Cd^2+^ toxicity at this time. In contrast, only about 10% of the cells transformed with the KO construct survived, whether survival was measured by number of motile cells or by cell counts, which is consistent with the production of *sh*RNA for *KIN5* knockdown by 12 h exposure to 0.5 µg/ml Cd^2+^. We attribute lethality mainly to the near absence of Kin5 in the cell, rather than to Cd^2+^ toxicity under these conditions. Lower concentrations of Cd^2+^ did not induce *sh*RNA for up to 24 h. There is a window of Cd^2+^ concentration and time of exposure that maximizes *sh*RNA production and minimizes Cd^2+^ toxicity. For essential proteins, like Kin5, it is important to demonstrate survival in control populations with the Cd^2+^ conditions chosen. Therefore, we used 0.5 µg/ml Cd^2+^and 12 h exposure as standard conditions, but flexibility should be possible.

### *KIN5* knockdown phenotype and Kin5 function in intraciliary transport

The anterograde transport of materials within many cilia and flagella is powered by at least two different motor complexes; a heterotrimeric kinesin-2 and a homodimeric kinesin-2. In *Tetrahymena*, Kin1 and Kin2 are motors of the heterotrimeric kinesin-2 whose individual knockout has little effect, but whose combined knockout leads to a resorption of cilia [6]. This suggests that, like *Chlamydomonas* Fla10 and Fla10h [5], the principal cargos of the Kin1/Kin2 motor complex are probably axonemal precursors, such as radial spoke proteins, necessary for the assembly and maintenance of the 9+2 cilium.

Kin5, the presumed homodimeric kinesin-2 characterized previously [7], is similar to Kin1/Kin2 in that the knockdown of this molecule results in its disappearance from cilia and eventual lethality. But, unlike the *kin1/kin2* knockout phenotype, the *KIN5* knockdown evidently does not cause ciliary resorption. Although forward progression is affected, cilia generally remain motile, until just before cell death. The disappearance implies that Kin5-based transport turns over recurrently to bring new motors, and presumably their cargos, into the cilium. Furthermore, the cargo transported by Kin5 cannot be critical for the maintenance of ciliary structure, except for perhaps at the cilium tip, or for motility, as demonstrated when cells are deciliated after knockdown. When RNA*i* for *KIN5* is induced, even though *KIN5* message remains, the cell regrowing the cilia end up with little or no Kin5. These cilia are motile. In *Tetrahymena*, Kin5 must at least in part be involved in a pathway of intraciliary transport where the cargo must presumably not include the axonemal precursors necessary for the building of the cilium.

In *C. elegans*, unlike *Tetrahymena*, the orthologous homodimeric kinesin-2, Osm3, and the heterotrimeric kinesin are redundant in building the cilia-based sensillum body [10], while Osm3 alone is the IFT transporter necessary to build the distal ends [9]. Presumably, membrane receptors are enhanced at the distal ends of invertebrate sensilla. The mammalian homologue of Kin5, Kif17, is endogenously expressed in the ciliary layer of olfactory neurons and in MDCK cell cilia. Coimmunoprecipitation suggests that Kif17 complexes with olfactory cyclic nucleotide-gated channel (CNG) subunits in olfactory epithelium, implying that Kif17 is the motor for CNG intraciliary transport. CNG channels transfected into MDCK cells are transported into their primary cilia [12]. Similar to *Tetrahymena*, but unlike *C. elegans*, transfection with dominant negative Kif3a results in complete loss of cilia, whereas transfection with dominant negative Kif17 does not produce ciliary resorption. CNG channels are clustered at the distal ends of olfactory cilia [24], suggesting that like Osm3, Kif17 is particularly important in transporting receptors to the distal ends of the cilia. In this regard, knockdown of *KIN5* may produce some ciliary shortening because Kin5 is necessary to build the mature cilia tip. However, this remains to be quantitated and the kinetics of Kin5 synthesis and turnover within the cilium remain to be defined.

### Gef1 is a Kin5 cargo

One viable hypothesis is that, like Kif17, and perhaps Osm3, Kin5 transports specific membrane proteins such as guanylyl cyclase [1] into the *Tetrahymena* cilium. Gef1 could be a cargo of Kin5 that facilitated concentration of targeted membrane receptors. Based on immunolocalization and sequence homology, Gef1 is a PH domain-containing GEF that is itself probably a ciliary membrane peripheral protein. Gef1 and Kin5 coimmunoprecipitate and in untransformed, uninduced or Inv2 cells induced with Cd^2+^, Gef1 and Kin5 localize together along the cilia. When cells are deciliated in the absence of *KIN5 sh*RNA induction, Gef1 and Kin5 reappear in a similar punctate pattern as new cilia grow. However, when cilia are examined after induction of *KIN5 sh*RNA so that Kin5 is no longer seen, Gef1 is still found along the ciliary membrane. When these cells are deciliated, cilia regrow without Gef1. These results suggest that Gef1 is released from the Kin5 transport apparatus to become resident in the ciliary membrane independent of further transport. Gef1 remains to be fully characterized, but a GEF is also found resident in the connecting cilium of mammalian photoreceptors as well as in ciliated epithelial tissues [25], [26] and in primary cilia [27]. GEFs interact with G-proteins involved in protein import into specialized cellular compartments, for example in nuclear import [28] Similarly, these results might imply that a specific GEF, Gef1 for *Tetrahymena*, would be localized along the ciliary membrane, where it might operate to facilitate release of other cargo, particularly membrane channels and receptors, imported into the cilium.

## Materials and Methods

### Plasmid Construction & Biolistic Transformation

20 µg of pMTT1-BICH3 (obtained from Dr. Martin Gorovsky, Univ. of Rochester, NY) was digested with HindIII and BamHI and resolved on a 1% low-melt agarose (Fisher Scientific, Pittsburgh, PA). The ∼6.0 KB vector was gel isolated away from the ∼1.5 KB of IAG48[G1] sequence using the Qiaquick Gel Isolation Kit (Qiagen, Valencia, CA) in a final elution volume of 50 µl. 1 µg of pK5KOAs.40s sense (GATCCCTTGACGCCACAAAAAAGATTCAAGAGATCTTTTTGTGGCGTCAAGTGAA) and 1 µg of pK5KOAs.40a anti-sense (GGAACTGCGGTGTTTTTTCTAAGTTCTCTAGAAAAACACCGCAGTTCACTTTCGA) oligonucleotides were mixed together in a 50 µl volume, heated at 95°C for 2 min, and incubated at 37°C for 2.5 h. A similar reaction was done for the inverse control using pInv2s sense (GATCCAGAAAAAACACCGCAGTTCTTCAAGAGAGAACTGCGGTGTTTTTTCTTGAA) and pInv2a anti-sense (GTCTTTTTTGTGGCGTCAAGAAGTTCTCTCTTGACGCCACAAAAAAGAACTTTCGA) oligonucleotides. 5 µl of the oligonucleotide mix were ligated with 5 µl of pMTT1-BICH3 (HindIII/BamHI digested and gel isolated to remove *IAG48[G1]*). CaCl_2_ competent *E coli* (DH5α) were transformed with the ligation mix. After plasmid minipreps (Qiagen), restriction digests and PCR were used to verify correct insertion.

20 µg of each vector was digested with KpnI/SacII (New England Biolabs) and phenol∶chloroform purified. 4 µl of the digest were mixed with 40 µl of prepared gold particles for biolistic transformation of *T. thermophila* CU522. After the shootings using the basic biolistic transformation protocol [29] and the Model PDS-1000/He Biolistic Particle Delivery System (Biorad, Hercules, CA), the cells were resuspended in 50 ml 2× proteose peptone (PP) plus 1× anti-mycotic mix (Gibco, Grand Island, NY). After incubation at room temperature for 2 h, paclitaxel (LC Laboratories, Woburn, MA) was added to a concentration of 20 µM. After 3 days at 25°C, a small aliquot was cultured into a 5 ml tube with 2XPP+20 µM paclitaxel. Every 3–4 days, ∼20 µl of cells were recultured into 5 ml 2XPP media containing 20 µM paclitaxel in order to complete phenotypic assortment. After a period of a few months, they were cultured into 2XPP media without any paclitaxel, maintained for at least two weeks and recultured by similar methods to allow for complete phenotypic assortment. They were then inoculated into 2XPP media containing 20 µM paclitaxel and grown overnight. Single-cells were isolated in drops and then transferred to 2 ml 2XPP with 1× anti-mycotic solution and grown to produce small clonal colonies for 3 days to select for cells that had completed phenotypic assortment with respect to the K5KO construct. Integration of the appropriate constructs was verified by PCR using the following primers: BTU1.a: TGGTTTAGCTGACCGATTCAG; MTT1.s: GCTGCTCAAAACATAGCTCATTC. The reaction conditions were performed on the Geneamp PCR System 2400 (Perkin Elmer, Boston, MA) using 1 µl of genomic DNA as follows: 95°C – 2 min; 95°C – 45 sec, 57°C – 1 min, 72°C – 1 min for 35 cycles; 72°C – 10 min; 4°C hold. 5 µl of the reaction products were resolved on a 1% agarose gel. Cells incorporating the RNA*i* construct were designated K5KOAs.40 (KO cells); cells incorporating the inverse construct were Inv2 cells.

### Cell Growth and Induction

KO and Inv2 cells were grown in 15 ml 2XPP for two days, spun down, resuspended in 5 ml starvation media (10 mM Tris, pH 7.5), incubated overnight without shaking, and diluted to 2.0×10^4^ cells/ ml. CdCl_2_ was added to a final concentration, either in a range from 0 to 0.5 µg/ml or 5.0 µg /ml. Aliquots of motile cells were studied. For ciliary regrowth experiments, deciliation was performed by shear. Cells were routinely examined microscopically for the presence of motility and cilia, and then placed in new 2XPP. Return of motility and beating cilia were monitored for up to 2 h.

### Cell Motility & Survival

Cell survival was measured by counting the cells in the motile population by hemocytometer. One to three readings were taken at each time point and a percentage survival calculated by comparison to t = 0. To ensure that the populations were composed of viable cells, motility was measured as the number of cell crossings at a pre-determined line in a one minute-time period. One to four readings were taken at t = 0 and every four hrs up to 24 hrs and calculated as a percentage corresponding to t = 0.

### RNA Isolation & RT PCR

Total RNA was isolated from equal number of cells at various time points using an RNeasy mini prep kit (Qiagen). Afterwards, 1 µl was used as template for the One-Step RT PCR kit (Qiagen) using either *KIN5* primers (tkin5.40s CCAGCAGCATAAGCTATGG; tkin5.40a ATGAAGACTGTTGCCGCCACC) or *PGM1* primers (PGM3s AAAAGGTTAGTGGTTGTTAAGG; PGM3a CTTGTGTAAATCATACTTTATTT) in a total reaction volume of 25 µl. The reaction conditions were performed on the Geneamp PCR System 2400 (Perkin Elmer) as follows: 50°C – 30 min, 95°C – 15 min; 95°C – 45 sec, 57°C – 1 min, 72°C – 1 min for 35 cycles; 72°C – 10 min; 4°C hold. 5 µl of the reactions were resolved on a 1% agarose gel. Within the linear range of product production as described by the kit, the amount of product produced is at least qualitatively proportional to the amount of message, which was confirmed by gel analysis of repeated multiple replications of the PCR experiments.

### Immunofluorescence

*T. thermophila* K5KOAs.40 and Inv2cells were incubated at 0.5 µg/ml CdCl_2_, spun down at different time points and resuspended in PHEM buffer (50 mM PIPES 50 mM HEPES 1 mM EGTA 2 mM MgS04) and an equal volume of Buffer A (PHEM buffer +4% paraformaldehyde +1% Triton X-100) containing 2 mM AMP-PNP (Sigma-Aldrich). After incubating for 5 min at room temperature, 1/20 volume of 10% Triton X-100 was added for 30 min. The cells were spun down (1100 g for 3 min) and washed 2× with TBST (10 mM Tris^-^HCl, pH 8.0 150 mM NaCl 0.05% Tween-20). For immunofluorescence microscopy, cells were incubated in the primary antibody for Kin5 [7], acetylated tubulin (Sigma-Aldrich), or Gef1 [20] for 15 min, washed two times in Buffer B (10 mM Tris^-^HCl pH 8.0, 150 mM NaCl, 0.05% Tween-20, 3% bovine serum albumin, 5 mM CaCl_2_), incubated with Cy3 or Cy5 secondary antibody (Jackson Lab, West Grove, PA) at 1∶100 dilution for 15 min and washed once with Buffer B. Controls omitted primary antibody incubation. Gef1 is a ciliary protein defined by a rabbit polyclonal antibody (Gef1 Ab) based on a peptide sequence (IQLMGRFDLDEEKDT) from the PH domain of a cloned *Paramecium* ciliary protein, PSec7 [20] probably a guanine nucleotide exchange factor (GEF1). A Scanalytics EPR deconvolution system (Scanalytics Inc., Fairfax, VA) [30] was employed for 3D reconstruction. Matched images were subjected to pseudocoloring for comparison of localizations. For critical analysis, the gain on the red channel was set to saturate the cell cytoplasm and individual cilia were selected. Colocalization in red and green images of the same cilium was compared by offsetting the images slightly [7].

### Western Blotting for Kin5 and Gef1

For western blotting, cilia fractions were prepared by shear or by dibucaine deciliation. After pelleting by centrifugation, membrane/matrix and axoneme fractions were prepared by demembranation in Triton ×100. Ciliary membranes were precipitated from the Triton supernate with 10%TCA and pelleted at 16000 g for 10 min. Normally, 15–20 µl samples derived from diluted packed cells calculated as about 10^4^ *Tetrahymena* cells or cell equivalents (from an original sample containing 10^6^ cells/ml) were used. Samples were run on a 7.5% SDS-PAGE gel and transferred to a nitrocellulose membrane (250 mA, 4 h) and blocked with blotto (10 mM Tris^-^HCl, pH 8.0, 150 mM NaCl, 0.05% TWEEN 20, 2 mM sodium azide, 2% non-fat milk) for one hour. The membrane was incubated overnight in blotto+affinity-purified K5T1 antibody (1∶100) or Gef1 Ab (1∶500). The membrane was washed three times in TBST (10 mM Tris^-^HCl, pH 8.0, 150 mM NaCl, 0.05% TWEEN 20, 2 mM sodium azide) for 5 min each and incubated with anti-rabbit secondary antibody (1∶4000 in blotto) conjugated with alkaline phosphatase (Sigma-Aldrich). After washing in TBST for 5 min each, the membrane was exposed to NBT/BCIP (KPL, Gaithersburg, MD).

### Coimmunoprecipitation

To ensure clean results with protein immunoprecipitation, a mucocyst-less strain of *T. thermophila* (SB255) was employed. 15 ml of *T. thermophila* SB255 at stationary phase were spun down (1100 g for 3 min) and resuspended in 1.0 ml lysis buffer (0.15 M NaCl, 1% Triton-X-100, 50 mM Tris). After incubation on ice for 30 min, the tube was spun (13200 RPM for 1 min) in a table-top centrifuge to remove cell debris. 100 µl of K5T1 antibody or Gef1 antibody was added and incubated at 4°C with gentle shaking overnight. 100 µl of Protein-A coated sepharose beads (10% v/v) were added and incubated at 4°C for one h. The tube was centrifuged (13200 RPM for 1 min) to pellet the beads which were washed twice with 350 µl lysis buffer and resuspended in 50 µl lysis buffer. After brief vortexing, 8 µl was used for Western blot with the opposite antibody (K5T1 Ab used for the Gef1 immunoprecipitate and the Gef1 Ab for the K5T1 immunoprecipitate).
